# Ligand recognition and signal transduction by lectin receptor-like kinases in plant immunity

**DOI:** 10.3389/fpls.2023.1201805

**Published:** 2023-06-15

**Authors:** Lu Liu, Jun Liu, Ning Xu

**Affiliations:** State Key Laboratory of Agrobiotechnology and MOA Key Laboratory for Monitoring and Green Management of Crop Pests, China Agricultural University, Beijing, China

**Keywords:** plant, receptor-like kinases, lectin receptor-like kinases, ligand recognition, immunity

## Abstract

Lectin receptor-like kinases (LecRKs) locate on the cell membrane and play diverse roles in perceiving environmental factors in higher plants. Studies have demonstrated that LecRKs are involved in plant development and response to abiotic and biotic stresses. In this review, we summarize the identified ligands of LecRKs in *Arabidopsis*, including extracellular purine (eATP), extracellular pyridine (eNAD^+^), extracellular NAD^+^ phosphate (eNADP^+^) and extracellular fatty acids (such as 3-hydroxydecanoic acid). We also discussed the posttranslational modification of these receptors in plant innate immunity and the perspectives of future research on plant LecRKs.

## Introduction

Plants constantly face challenges from bacteria, fungi, viruses, oomycetes, and nematodes. To fight against these threats, plants have evolved a sophisticated innate immune system. Conserved pathogen-/damage-/microbe-/herbivore-associated molecular patterns (PAMPs/DAMPs/MAMPs/HAMPs) are recognized by cell surface-localized pattern recognition receptors (PRRs), leading to the pattern-triggered immunity (PTI) responses (Jones and Dangl, 2006; Tanaka and Heil, 2021; Snoeck et al., 2022). Pathogens can secrete virulence effectors into plant cells through delivery devices, such as the type III secretion system (T3SS) in bacteria, haustoria in fungi and oomycetes, and stylets in aphids and nematodes, thereby inhibiting PTI responses and promoting infection (Dangl et al., 2013). To counteract the infection, the intracellular nucleotide-binding leucine-rich repeat receptors (NLRs) of plants recognize the cognate pathogen effectors, in many cases triggering a strong immune response, such as hypersensitive response (HR), referring to effector-triggered immunity (ETI) (Jones and Dangl, 2006). Recently, studies have demonstrated that PTI and ETI are not two independent immune pathways. In contrast, ETI enhances PTI immune response, and PTI, on the other hand, reinforces ETI-induced cell death (Ngou et al., 2021; Tian et al., 2021; Ngou et al., 2022).

PRRs, a group of cell surface receptors, consist of two different types of cell surface-localized proteins, receptor-like kinases (RLKs) and receptor-like proteins (RLPs) (Couto and Zipfel, 2016). RLKs possess three functional domains, including an *N*-terminal variable ligand-binding ectodomain, a single-pass transmembrane domain, and an intracellular kinase domain (Walker, 1994). Unlike RLKs, RLPs lack the intracellular kinase domain but have a short cytosolic tail (Jeong et al., 1999). The typical extracellular ligand-binding domains of RLKs and RLPs include the leucine-rich repeat (LRR), lysine motif (LysM), lectin domain, and an epidermal growth factor (EGF)-like domain. However, some RLKs, such as BAK1 and SERK1, lack functional extracellular domains. Instead, they have the functional kinase domains and act as coreceptors or scaffold proteins for the typical RLKs or RLPs that possess extracellular domains (Li et al., 2002; Stegmann et al., 2017; Liang and Zhou, 2018).

Lectin RLK (LecRK) is a large subfamily of RLKs and its *N*-terminal lectin domain reversibly binds to carbohydrates (Bouwmeester and Govers, 2009). Based on the characteristics of extracellular lectin domains, LecRKs are classified into three types: G-type, L-type, and C-type lectins (Vaid et al., 2013). The number of LecRKs varies dramatically among different species (**Table 1**). G-type LecRKs contain an α-mannose binding bulb lectin domain, an S-locus glycoprotein domain (SLG), and a PAN and/or Epidermal Growth Factor (EGF) domain (Vaid et al., 2013; Sun et al., 2020). L-type LecRKs contain a legume-lectin domain with a typical β-sandwich structure (Bellande et al., 2017). In plants, the number of C-type LecRKs, which are predicted to be calcium-dependent kinases, is quite small and their function remains unclear (Sun et al., 2020). The central transmembrane domain of LecRKs usually consists of approximately 18-25 amino acid residues. Kinase domains generally consist of 250-300 amino acid residues and contain various conserved phosphorylation sites that are responsible for transducing external signals to downstream pathways (Morillo and Tax, 2006).

**Table 1:** The number of LecRK genes in plant.

Organism	L-type	G-type	C-type	Total	Reference
*Arabidopsis thaliana*	45	40	1	86	(Bouwmeester and Govers, 2009)
*Cerasus humilis*	43	125	2	170	(Han et al., 2021)
*Glycine max*	64	123	2	189	(Liu et al., 2018b)
*Oryza sativa*	72	100	1	173	(Vaid et al., 2012)
*Prunus avium*	50	110	1	161	(Sun et al., 2022)
*Populus trichocarpa*	50	180	1	231	(Yang et al., 2016)
*Setaria italica*	53	59	1	113	(Zhao et al., 2016)
*Solanum lycopersicum*	22	38	1	61	(Wang et al., 2015c)
*Solanum tuberosum*	26	85	2	113	(Zhang et al., 2020a)
*Saccharum officinarum*	160	266	3	429	(Wang et al., 2021)
*Triticum aestivum*	84	177	2	263	(Shumayla et al., 2016)
*Vigna radiata*	34	38	1	73	(Singh et al., 2021)
*Zea mays*	48	46	1	95	(Yang et al., 2016)

Several excellent reviews have described the structures and functions of LecRKs in various abiotic and biotic processes, and in the process of plant development (Vaid et al., 2012; Singh and Zimmerli, 2013; Vaid et al., 2013; Sun et al., 2020). Wang et al. performed a systematic functional analysis to evaluate phenotypic changes in *Arabidopsis* LecRK T-DNA insertion lines in response to pathogen infection and abiotic stress treatment (Wang et al., 2014). We summarized the recent research progress of LecRKs in **Table 2**. In this review, we will mainly focus on summarizing the latest findings in ligand recognition, posttranslational modification, and natural variation of LecRKs.

**Table 2:** The function of LecRKs in different plants.

Name	Type	Organism	Ligand	Function	References
Biotic processes					
LecRK-I.5	L	*Arabidopsis thaliana*	eATP	Recoginze eATP and defense against *Pseudomonas syringae*	(Pham et al., 2020)
LecRK-I.8	L	*Arabidopsis thaliana*	eNAD^+^	Recoginze eNAD^+^ and Perceive *Pieris brassicae* eggs	(Wang et al., 2017; Gouhier-Darimont et al., 2019)
LecRK-I.9	L	*Arabidopsis thaliana*	eATP	Recoginze eATP and enhance plant resistance against multiple pathogens	(Bouwmeester et al., 2014; Choi et al., 2014a; Balagué et al., 2017; Jewell et al., 2022)
LecRK-V.5	L	*Arabidopsis thaliana*	–	Response to *Pseudomonas syringae* pv*. tomato* DC3000 and *Pectobacterium carotovorum*	(Arnaud et al., 2012; Desclos-Theveniau et al., 2012)
LecRK-VI.2	L	*Arabidopsis thaliana*	eNAD^+^ and NADP^+^	Recoginze eNAD^+^ and NADP^+^, Resistance against *Pseudomonas syringae*	(Singh et al., 2012; Wang et al., 2019)
LecRK-IX.2	L	*Arabidopsis thaliana*	–	Defense against *Phytophtora* and *Pseudomonas syringae*	(Wang et al., 2015b; Luo et al., 2017; Xu et al., 2020)
LecRK-V	L	*Haynaldia villosa*	–	Defense against powdery mildew	(Wang et al., 2018)
SlLecRK1	L	*Solanum lycopersicum*	–	Increase the tomato resistance against *Fusarium oxysporum* f. sp. *radicis-lycopersici*	(Yue et al., 2022)
StLecRK-IV.1	L	*Solanum tuberosum*	–	Negatively regulate plant resistance to *Phytophthora infestans*	(Guo et al., 2022)
LORE	G	*Arabidopsis thaliana*	3-OH-C10:0	Recoginze 3-OH-C10:0 and interact with effector HopAO1	(Ranf et al., 2015; Kutschera et al., 2019; Luo et al., 2020)
Pi-d2	G	*Oryza sativa*	–	Resistance against *Magnaporthe grisea* strain ZB15	(Chen et al., 2006)
PtLecRK	L	*Sphaerulina musiva*		Resistance against *Sphaerulina musiva*	(Muchero et al., 2018)
PtLecRLK1	G	*Populus trichocarpa*	–	Participate in *Populus*–*Laccaria bicolor* symbiotic interactions	(Labbé et al., 2019)
Abiotic processes					
LecRK-VII.1	L	*Arabidopsis thaliana*	–	Regulate plant tolerance to salt stress	(Zhang et al., 2016)
LecRK-b2	L	*Arabidopsis thaliana*	–	Response to salt and osmotic stress	(Deng et al., 2009)
PnLecRLK1	L	*Pohlia nutans*	–	Enhance *Arabidopsis* chilling-stress tolerance	(Liu et al., 2016)
GmLecRLK	G	*Glycine max*	–	Improve the tolerance of soybean to salt stress	(Zhang et al., 2022)
Development					
LecRK-VIII.2	L	*Arabidopsis thaliana*	–	Regulate seed yield and source–sink relationship	(Xiao et al., 2021)
AP1	L	*Oryza sativa*	–	Promote starch accumulation during rice pollen maturation	(He et al., 2021)
OsDAF1	L	*Oryza sativa*	–	Regulate pollen aperture patterning formation	(Zhang et al., 2020b)
OsLecRK‐S.7	L	*Oryza sativa*	–	Regulate pollen development and male fertility	(Peng et al., 2020)
TaLecRK-IV.1	L	*Triticum aestivum*	–	Promote wheat height	(Saidou and Zhang, 2022)
OslecRK	G	*Oryza sativa*	–	Promote seed germination and enhance seed vigor	(Cheng et al., 2013)
SlG-LecRK-II.9	G	*Solanum lycopersicum*	–	Promote pollen grain development	(Micol-Ponce et al., 2023)

## Ligand perception by LecRKs

LecRKs are widely distributed in plant kingdoms, but interestingly they have no orthologs in yeast and human genomes (Navarro-Gochicoa et al., 2003). L-type LecRKs have a conserved hydrophobic cavity that can bind hydrophobic ligands like monosaccharides (glucose/fucose/mannose) or polypeptides, while G-type LecRKs exhibit a strong high binding affinity to α-D mannose. Due to the diversity of LecRKs and their target carbohydrates, it is difficult to identify the ligand-receptor interaction between them (Bellande et al., 2017). To date, only four ligands (eATP, eNAD^+^, eNADP^+^, and 3-OH-C10:0) of LecRKs have been identified.

### ATP

ATP is not only an essential energy currency in nature, but also acts as an enzyme cofactor. Moreover, ATP is released into the extracellular matrix after wounding or other environmental stimulation (Kim et al., 2006; Khakh and Burnstock, 2009; Dark et al., 2011; Ramachandran et al., 2019). At present, eATP signaling has been widely studied in animals. Most mammals contain two families of ATP receptors, seven P2X receptors and eight P2Y receptors. P2X receptors are ion channels on the plasma membrane and are activated by the binding of eATP (Khakh and North, 2006). P2Y receptors are a family of metabotropic receptors that couple to intracellular second-messenger systems through heteromeric G-proteins (Van Der Giet et al., 2002). These receptors have diverse temporal and spatial expression patterns and respond differently to individual nucleotide ligands (Lustig et al., 1993; Webb et al., 1993; Ralevic and Burnstock, 1998; Abbracchio et al., 2006).

**Figure 1:**
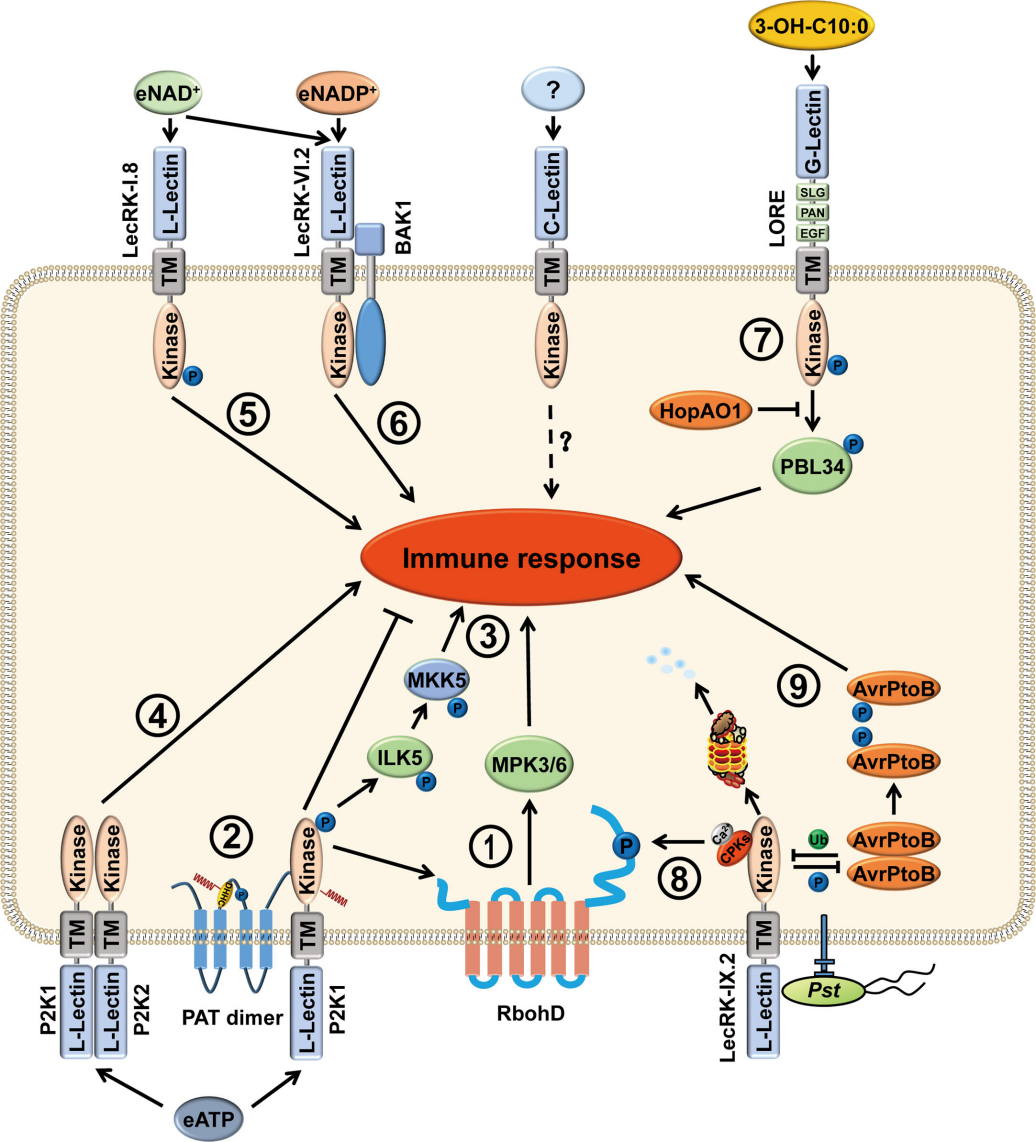
Functions of LecRKs in stress responses of plants. LecRKs are divided into three types, L-type, G-type and C-type, based on the extracellular lectin domain in plants. LecRKs can recognize specific extracellular signaling molecules and transduce signals into the cell, thus activating the plant immune response. (1) P2K1 (LecRK-I.9) perceives eATP and then phosphorylates RbohD and MPK3/6, causing increased production of ROS and stomatal closure to fight against pathogen infection. (2) In addition, P2K1 directly interacts with and phosphorylates PATs to activate their S-acylation ability. Activated PATs S-acylate P2K1 to inhibit the immune response and protect plant growth. (3) In the presence of eATP, P2K1 interacts with and phosphorylates ILK5. Then, ILK5 interacts with and phosphorylates the MKK5, leading to an activated immune response. (4) Upon eATP treatment, P2K1 and P2K2 (LecRK-I.5) interact with each other and may function as part of a heteromeric complex. (5) LecRK-I.8 can perceive eNAD^+^ and activate the plant immune response. (6) LecRK-VI.2 is a potential receptor for eNAD^+^ and eNADP^+^. Furthermore, LecRK-VI.2/BAK1 complex mediates systemic acquired resistance that is triggered by eNAD^+^. (7) G-type LecRK LONE is the receptor of 3-OH-C10:0. After perception of 3-OH-C10:0, the phosphorylation level of LORE Y600 is elevated and trans-phosphorylates PBL34 at T306 and T310. Phosphorylated PBL34 dissociates from LORE, activating immune response. However, the bacterial effector HopAO1 targets LORE Y600 and dephosphorylates the tyrosine-phosphorylated Y600 to suppress immune response. (8) When perceiving pathogen infection, LecRK-IX.2 recruits CPKs to phosphorylate RbohD, resulting in PTI activation, ROS production, and the subsequent ROS triggered SA accumulation. (9) Moreover, the bacterial effector AvrPtoB ubiquitinates LecRK-IX.2 to degrade LecRK-IX.2, thus suppressing LecRK-IX.2-mediated PTI. However, LecRK-IX.2 phosphorylates AvrPtoB at S335 which leads to AvrPtoB dimer dissociation and reduces its virulence.

In contrast to mammals, the function of eATP in plants is poorly understood. During growth and in response to various biotic and abiotic stimuli, ATP can be released into the extracellular matrix. eATP plays fundamental roles in mediating plant defense against pathogens and herbivores, such as the production of reactive oxygen species (ROS), elevation of cellular Ca^2+^ concentration, activation of mitogen-activated protein kinase (MPK) phosphorylation, and indolic glucosinolate pathway (**Figure 1**) (Demidchik et al., 2003; Roux and Steinebrunner, 2007; Tanaka et al., 2010; Liu et al., 2012; Feng et al., 2015; Cho et al., 2017; Jewell et al., 2022). However, plant genomes do not contain potential orthologs of animal P2X and P2Y receptors (Fountain et al., 2008; Tanaka et al., 2010). Nevertheless, eATP did induce NADPH oxidase-mediated accumulation of superoxide (O_2_^-^), indicating that plants must possess a receptor for eATP (Song et al., 2006). Until 2014, the first eATP receptor, the DOes not Respond to Nucleotides 1 (DORN1), was identified in *Arabidopsis* by forward genetic screening approach (Choi et al., 2014a). DORN1 is an L-type lectin receptor kinase that binds ATP with high affinity (dissociation constant of 45.7 ± 3.1 nM). In addition, eight ethylmethanesulfonate (EMS) mutants and two T-DNA insertion lines, which are distributed in the entire gene of *DORN1*, including the extracellular lectin domain, the transmembrane domain and the intracellular serine/threonine kinase domain, all showed defects in eATP-induced calcium influx (Choi et al., 2014a; Choi et al., 2014b). Therefore, DORN1 is a bona fide receptor for eATP.

*Arabidopsis* DORN1, also termed P2K1 or LecRK-I.9, belongs to the L-type LecRK subfamily, which contains 45 members. Most LecRK-encoded genes are differentially expressed in various tissues and at different developmental stages. Their expression can be induced by various elicitors and pathogen infections (Bouwmeester and Govers, 2009). *dorn1* mutants completely lose the ability to respond to eATP in young seedlings. However, the diverse expression patterns of different LecRKs suggest the possibility that there are other LecRKs involved in the eATP-activated signaling pathway (Choi et al., 2014a; Choi et al., 2014b). In *Arabidopsis*, L-type LecRK family is divided into nine subclades; P2K1 belongs to clade I which consists of 11 members. Recently, by screening all members of the LecRK clade I, only P2K2 (LecRK-I.5) was able to partially restore the Ca^2+^ response in *p2k1* mutant plants. Further experiments confirmed that P2K2 is a second purinergic receptor. P2K1 and P2K2 showed self-association and formed heterodimer with each other upon ATP treatment (Chen et al., 2017; Pham et al., 2020) The fact that the low expression of *P2K2* and activation of P2K1 leads to transphosphorylation of P2K2 likely explains why P2K2 was not identified in the initial eATP response deficient mutant screening (Pham et al., 2020).

A previous study showed that by analyzing data from Genevestigator V3 web, different *Arabidopsis LecRK* genes had different expression profiles in response to hormone treatment, abiotic stress, elicitor treatment and pathogen infection (Bouwmeester and Govers, 2009). We analyzed and visualized the relative expression patterns of 45 L-type *AtlLecRKs* under both cold (4°C) and heat (38°C) stresses using Plant eFP database (**Figure S1**). Most *AtlLecRKs* showed obvious changes at 12 h after cold treatment and 1h after heat treatment. *P2K1* did not show obvious changes after temperature treatment, however, *P2K2* could be suppressed by cold treatment and induced by heat. Therefore, by changing specific growth conditions, such as high or low temperature, it may be possible to identify additional eATP receptors in plants in the future.

### NAD^+^

NAD^+^ and NAD phosphate (NADP^+^) are electron carriers in most of metabolic reactions. In mammalian cells, NAD^+^ is released into the extracellular space upon cell death and inflammation, which could potentially activate immunity, but whether there is an eNAD^+^-recognition receptor remains elusive (Billington et al., 2006). In *Arabidopsis*, many experiments have revealed that NAD^+^ and NADP^+^ are released into the extracellular compartment during infection by the bacterial pathogens. NAD^+^ or NADP^+^ release leads to the activation of salicylic acid (SA) signaling pathway and the accumulation of ROS resulting in disease resistance (Zhang and Mou, 2009; Pétriacq et al., 2012; Zhang and Mou, 2012; Pétriacq et al., 2016). In addition, exogenously applied NAD^+^ moves systemically and induces systemic acquired resistance (SAR) (Wang et al., 2019). These studies suggest that there is an NAD^+^ or NADP^+^ receptor to sense these signals in *Arabidopsis*.

In 2017, Wang et al. analyzed the transcriptomic changes in *Arabidopsis* after eNAD^+^ treatments. The microarray data indicated that the expression of some receptor kinases was induced by NAD^+^. In the receptor kinase mutant *lecrk I.8*, NAD^+^-induced *PR1* expression was significantly decreased and disease resistance to *P. syringae* pv. *maculicola* (*Psm*) ES4326 was largely compromised (**Figure 1**) (Wang et al., 2017). LecRK-I.8 specifically binds to NAD^+^, but not to NADP^+^, ATP, ADP, or AMP. In contrast, P2K1, LecRK-I.3, and LecRK-I.6 do not bind to NAD^+^. Moreover, although *LecRK-I.8* null mutants do not respond to low concentration of NAD^+^, it does have response to high concentrations of NAD^+^, including induced *Pathogenesis-Related gene* (*PR*) expression and enhanced disease resistance. These data suggest that LecRK-I.8 is not the only receptor that perceives eNAD^+^ (Wang et al., 2017). In addition, LecRK-I.8 is also a potential cell surface receptor for *Pieris brassicae* egg-derived elicitors, where loss of LecRK-I.8 significantly reduced the immune response to the egg extract, suggesting that there is a potential egg-derived ligand for LecRK-I.8 (Gouhier-Darimont et al., 2019).

Although LecRK-I.8 is a potential receptor for NAD^+^, it does not bind NADP^+^ (Wang et al., 2017). Later, the same group found that LecRK-VI.2 is a potential receptor for eNADP^+^, where NADP^+^-induced disease resistance is clearly reduced in *lecrk-VI.2* mutant*s*. The recombinant extracellular domain of LecRK-VI.2 (eLecRK-VI.2) exhibited a typical saturation curve in binding with ^32^P-NAD^+^. Unlabeled NAD^+^ and NADP^+^ compete for binding of ^32^p-NAD^+^, but unlabeled ATP cannot. However, the binding affinity of NADP^+^ with eLecRK-VI.2 is slightly higher than that of NAD^+^ (**Figure 1**) (Wang et al., 2019). Notably, exogenous NAD(P)^+^-induced systemic *PR1* transcription was only slightly reduced in *lecrk-VI.2* mutants, suggesting that there are other receptors that can sense NAD(P)^+^.

### Lipopolysaccharide and 3-OH-C10:0

Lipopolysaccharide (LPS), a major cell wall component of gram-negative bacteria, consists of three different functional moieties: lipophilic lipid A, a core oligosaccharide, and O-antigen (Ranf, 2016; Kagan, 2017). In mammals, LPS acts as MAMP and triggers strong immune responses, and the molecular mechanism for LPS recognition has been well studied. The event can be divided into intracellular and extracellular recognition in two different ways. In extracellular recognition, Toll-like receptor 4 (TLR4) forms a heterodimer with myeloid differentiation factor 2 (MD2) to recognize LPS and initiates intracellular signaling mediated by caspase-11 (Park et al., 2009; Kayagaki et al., 2011). Moreover, LPS-binding protein (LBP) and CD14 are also essential for LPS recognition (Tsukamoto et al., 2018). In intracellular recognition, human caspase-4 and its mouse homologue caspase-11 can directly bind to LPS and lipid A with high specificity and affinity, resulting in activation of caspases (Shi et al., 2014).

Compared to the studies of the responses to LPS in mammals, there are few studies of the mechanisms involved in LPS recognition and downstream signal transduction in plants. LPS-elicited defense-related responses include programmed cell death, nitric oxide (NO) production, *PR* gene expression, and ROS burst in plants (Zeidler et al., 2004; Desaki et al., 2006; Sun et al., 2012; Proietti et al., 2014; Shang-Guan et al., 2018). Using a forward-genetic approach to screen *Arabidopsis* mutants that are insensitive to LPS, Ranf et al. reported that a G-type LecRK LORE (lipooligosaccharide-specific reduced elicitation) is required for LPS -mediated PTI responses (Ranf et al., 2015). Moreover, they found that lipid A moiety is indispensable and sufficient for LORE-dependent sensing of LPS. Removal of the ester-linked acyl chains from lipid A abolishes its ability to trigger plant immunity (Ranf et al., 2015). In *Arabidopsis*, *miR393* regulates one L-type LecRK and two G-type LecRKs following the perception of bacterial LPS to initiate immunity and basal resistance (Djami-Tchatchou and Dubery, 2019). These results suggest that LPS and acylated Lipid A are essential for immune sensing in *Arabidopsis*.

However, the same group in 2019 found that medium-chain 3-hydroxy fatty acids (mc-3-OH-FAs) rather than LPS, produced during LPS biosynthesis, are sensed by LORE (**Figure 1**). mc-3-OH-FAs can be copurified with LPS and lipid A. mc-3-OH-FA-depleted LPS does not activate the immune responses (Kutschera et al., 2019). The immune strength elicited by mc-3-OH-FAs depends on the chain length and the specificity of hydroxylation. Free 3-OH-C10:0 is the strongest immune molecule and has a strong binding affinity with the LORE ectodomain. In addition, LORE can sense the (R)-3-hydroxyalkanoate precursors HAAs which are involved in bacterial surface dissemination and biofilm development (Schellenberger et al., 2021). These results indicate that plants can also utilize LecRK to perceive pathogens by sensing the conserved features of microbial metabolites.

## Modification of LecRKs

Protein phosphorylation and ubiquitination are two major posttranslational modifications in eukaryotes (Mithoe and Menke, 2018). These modifications play important roles in the turnover of proteins, signal transduction, and cellular homeostasis.

### Phosphorylation modification

#### Phosphorylation and autophosphorylation of LecRKs

Phosphorylation is required for signal transduction and for maintaining cellular homeostasis upon receptor complex activation. Previous studies have shown that phosphorylation regulation is critical for the function of protein kinase RLKs (Couto and Zipfel, 2016; Tang et al., 2017; Mithoe and Menke, 2018). PnLPK, a lectin-like protein kinase in *Populus nigra* var. *italica*, is the first identified LecRK in plants, and exhibits autophosphorylation activity on serine and threonine residues in the protein kinase domain but not on tyrosine residues (Nishiguchi et al., 2002). Further studies proved that the autophosphorylation of LecRKs plays important roles in signal transduction in *Arabidopsis* and rice. AtLecRK-VI.2 has autophosphorylation activity *in vitro* in the presence of divalent metal cations and positively modulates early bacterium-mediated MPK signaling (Singh et al., 2012; Singh et al., 2013). P2K1 has autophosphorylation activity and this activity is essential for ATP-induced plant responses (Choi et al., 2014a; Choi et al., 2014b). Mass spectrometry (MS) and biochemistry assays revealed that autophosphorylation of S391, S440, and S451 is critical for P2K1 kinase domain-mediated eATP recognition. Phosphomimetic S391D, S440D, and S451D mutants exhibited a significant increase in Ca^2+^ cyt concentration after ATP addition (Chen et al., 2017). Overexpression of LecRK-IX.2 leads to spontaneous cell death in *Arabidopsis* (Wang et al., 2015b). In contrast, three kinase-dead LecRK-IX.2 mutant variants, LecRK-IX.2^K379R^, LecRK-IX.2^K477E^, and LecRK-IX.2^D532N^, not only have no autophosphorylation activity, but also lost the ability to elicit cell death in *N. benthamiana* (Luo et al., 2017).

In addition to the serine/threonine phosphorylation, recent studies have demonstrated that the tyrosine phosphorylation of LRR-RLKs and LysM-RLKs is also essential for immune activation (Liu et al., 2018a; Perraki et al., 2018). Although plants do not have tyrosine kinases discovered in animals, many RLKs, including BAK1, have been shown to possess dual specificity kinases, auto-phosphorylating *in vitro* on serine/threonine as well as tyrosine residues (Perraki et al., 2018). Luo et al. showed that LORE is also a dual-specificity protein kinase (**Figure 1**) (Luo et al., 2020). Y600 in LORE is a conserved residue in many immunity-related RLK orthologs, including G-type lectin kinases, EFR, CERK1, and BAK1 (Macho et al., 2014; Liu et al., 2018a; Perraki et al., 2018; Luo et al., 2020). Substitution of Y600 with phenylalanine markedly reduces the kinase activity of LORE and LORE-mediated 3-OH-C10:0 recognition in *Arabidopsis*.

### The phosphorylation targets of LecRKs

In addition to autophosphorylation, LecRKs can also phosphorylate other proteins to transmit the signal to downstream pathways. In rice, a G-type LecRK OsPID2 is able to phosphorylate OsPUB15, a U-box/ARM repeat protein. Phosphorylated OsPUB15 exhibits an active E3 ligase activity. In addition, overexpression of OsPUB15 in rice leads to a spontaneous cell death phenotype as well as a constitutive activation of plant basal defense responses (Chen et al., 2006; Wang et al., 2015a). The L-type LecRK Salt Intolerance 1 (SIT1) phosphorylates MPK3/6 to mediate salt sensitivity and regulates ethylene homeostasis in rice (Li et al., 2014). In *Arabidopsis*, upon perception of eATP, P2K1 directly phosphorylates the NADPH oxidase RBOHD and the Raf-like MAP kinase kinase kinase INTEGRIN-LINKED KINASE 5 (ILK5), which subsequently elevates the production of ROS, induces stomatal closure and triggers innate immune response (**Figure 1**) (Chen et al., 2017; Kim et al., 2023). Although LecRK-IX.2 interacts with RBOHD, it is unable to directly phosphorylate RBOHD. Instead, LecRK-IX.2 likely recruits the calcium-dependent protein kinases (CPKs) to phosphorylate and activate RBOHD, which leads to the ROS burst and the enhanced ROS-triggered SA biosynthesis (**Figure 1**) (Luo et al., 2017). Recent phosphoproteomics biochemical results showed that cGMP-dependent protein kinase is a potential substrate of PtLecRLK1 (Shrestha et al., 2023).

PRRs often trans-phosphorylate RLCKs to activate immune signaling from extracellular ligand perception into downstream signaling in plants (Monaghan et al., 2014; Liang and Zhou, 2018). Our recent study showed that LORE-mediated immune activation follows this rule. After perception of the ligand 3-OH-C10:0, LORE is autophosphorylated at Y600, which further transphosphorylates the receptor-like cytoplasmic kinases PBL34, PBL35, and PBL36 to activate plant immunity. However, *Pseudomonas syringe* pv. *tomato* (*Pst*) effector HopAO1, which is a phosphatase, targets LORE to dephosphorylate tyrosine-phosphorylated Y600 and suppresses immune responses (Luo et al., 2020). Whether other LecRKs also rely on RLCKs to activate the immune signaling deserves further investigation.

### Ubiquitination and palmitoylation modifications

Ubiquitination plays an important role in maintaining cellular homeostasis and activating the immune receptor complex in plants. For example, *Arabidopsis* LRR-RLK FLAGELLIN-SENSING 2 (FLS2) can be polyubiquitinated by two U-box E3 ligases, PUB12 and PUB13, to attenuate immune signaling upon flagellin recognition (Lu et al., 2011). The large-scale identification of ubiquitination sites in *Arabidopsis* reveals that over 100 protein kinases that are associated with or integrated into the plasma membrane are ubiquitinated, including L-type LecRKs, LecRK-VII.2 (K339), LecRK-VIII.1 (K374), RLK/LecRK-IV.1 (K350, K370), and a C-type lectin receptor kinase (K265, K278, K292) (Grubb et al., 2021). We recently found that *Pst* effector AvrPtoB, a 553-amino-acid protein with a functional E3 ligase domain, targets and degrades LecRK-IX.2 to suppress LecRK-IX.2-mediated PTI. In contrast, LecRK-IX.2 phosphorylates AvrPtoB at serine site 335 to interrupt AvrPtoB self-association and reduce its E3 ligase activity (**Figure 1**) (Xu et al., 2020). Very recently, OsPID2, a key protein in the resistance of rice to *Magnaporthe oryzae* strain ZB15, was shown to interact with U-box E3 ubiquitin ligase OsPIE3 (PID2-interacting E3). This interaction leads to the intracellular kinase domain of PID2 entering the nucleus and reducing blast disease resistance (Wang et al., 2023). These findings indicate that ubiquitination is important in maintaining the function of LecRKs.

Recently, LecRK S-acylation has been reported. S-acylation is a reversible and cycling posttranslational modification, in which cysteine residues are conjugated with a variety of acyl chains through a thioester bond; however, how to regulate this process is still poorly understood in plants. *Arabidopsis* plant encodes 24 S-acyl transferases (PATs) which contain a conserved Asp-His-His-Cys (DHHC) catalytic domain; however, the specific substrates of these AtPATs are barely identified (Batistic, 2012). A previous study has identified two proteins from the CLAVATA2 (CLV2)-like AtRLP subfamily and eight RLCK subfamily members that are S-acylated, indicating that S-acylation may play an important role in the LRR-RLK/AtRLP/RLCK signaling pathway (Hemsley et al., 2013). Although subsequent study suggests that juxta-membrane S-acylation of plant RLKs is likely fortuitous, S-acylation of FLS2 adjacent to the transmembrane domain is not required for FLS2-mediated immune signaling (Hurst et al., 2019). A recent study discovered that two DHHC-PAT proteins AtPAT5 and AtPAT9 negatively regulate the plant immune receptor P2K1 *via* S‐acylation. Upon perception of eATP, P2K1 is rapidly autophosphorylated, which then in turn phosphorylates PATs to activate PAT mediated S-acylation. Activation of PATs can S-acylate P2K1 to reduce P2K1 phosphorylation, dampen the immune response and protect growth (**Figure 1**) (Chen et al., 2021). These works highlight the important roles of S-acylation in plant innate immunity.

## Natural variation of LecRKs

Due to technical advances in genetic mapping, resequencing, and genome-wide association study (GWAS), some LecRKs have been identified to regulate many biological processes in plants. A recent GWAS using a set of 295 natural *Arabidopsis* accessions showed that LecRK-I.1, a close homolog of LecRK-I.8, is specifically involved in large white butterfly *Pieris brassicae* egg extract (EE)-triggered HR-like response (Groux et al., 2021). There are five most significant single nucleotide polymorphisms (SNPs) in the *LecRK-I.1* gene. One SNP is located in the carbohydrate-binding lectin-like domain. Two are located in the kinase domain, while the other two are silent mutations. Further analysis found that two main haplotypes of LecRK-I.1 segregated at the population level and the SNP in the kinase domain was significantly associated with the EE-triggered HR-like responses (Groux et al., 2021). Moreover, three novel QTLs were determined by genetic analysis of EE-induced HR-like cell death in 56 *Brassica rapa* accessions. These three QTLs include many candidate genes that are involved in plant immunity processes One QTL contains a cluster of *LecRK-I* genes, which are homologous genes of *AtLecRKI.1* Another QTL includes *LecRK-V.5*, which negatively modulates plant immunity against necrotrophic bacteria (Arnaud et al., 2012; Bassetti et al., 2022). Further fine-mapping of these identified QTLs will help to identify the key genes. By GWAS mapping of *ca*. 1,000 resequenced *Populus trichocarpa trees* individually challenged with an invasive fungal pathogen *Sphaerulina musiva*, one L-type LecRK was associated with resistance and one G-type LecRK was a susceptibility-associated locus. The L-type LecRK had a mutant frequency of 10% and predicted to have a high impact on protein translation. The G-type LecRK showed only two high-impact mutations at frequency 1.5% and 8.0%. Biochemical results demonstrated that both LecRKs lection domain bound to cell wall preparations of *S. musiva* (Muchero et al., 2018). Intriguingly, Lin et al. used another advanced technology named RLP/KSeq and successfully fine-mapped the SCR74 (an apoplastic effector of *Phytophthora infestans*) receptor to a 43-kbp *G-LecRK* locus in *Solanum microdontum* plants (Lin et al., 2020).

Recently, some LecRKs have been identified to respond to abiotic stress treatments. GWAS and epigenome wide association studies (EWAS) for the 60 *Populus tomentosa* hub genes identified that LecRK-VIII.2 is associated with chlorophyll content among different methylotypes (Zhou et al., 2023). Using in-field overwinter survival as a trait, another GWAS and population differentiation analysis in *Lotus japonicas* revealed that LjLecRK is required for non-acclimated freezing tolerance and haplotype dependent cold responses (Shah et al., 2020; Mustamin et al., 2023). As there is tremendous natural variation in LecRKs, more LecRKs may be identified by GWAS analysis combined with different biotic or abiotic treatments in the future.

## Conclusions and future perspectives

In the past decade, our knowledge about plant LecRKs has greatly advanced. Some new ligands have been found. Accordingly, new modifications and natural variations of LecRKs have been discovered. Although many LecRKs have been identified in plants, very few LecRKs have been functionally characterized. Of these LecRKs, most have been discovered in *Arabidopsis*. Future studies should explore the ligand and signal transduction of LecRKs in other plant species.

### Ligand identification

By forward genetic screen approach and transcriptomics, a few ligands of LecRKs have been identified. However, the highly variable lectin domains of LecRKs imply that a wide range of ligands have not been identified in plants. Self-molecules, such as eATP, eNAD^+^ and lipids from pathogens are known ligands that can be recognized by LecRKs. The current challenge is that although LecRKs have sugar-binding residues, they are poorly conserved and unlikely to bind monosaccharide molecules (Bouwmeester and Govers, 2009). 3-OH-C10:0 is hydrophobic ligand and the main egg-derived elicitors which can activate the expression of *PR1* in *Arabidopsis* are enriched in the fraction containing total egg lipids (Bruessow et al., 2010; Kutschera et al., 2019). This indicates that some LecRKs may recognize some hydrophobic compounds from insect egg. In addition, metabolomic analysis of apoplast wash fluid is a powerful tool for discovering novel ligands. Suspension cells can also be used to search for potential ligands after stress treatment. Some small signaling peptides have been identified from chitin-treated rice suspension cells combined with metabolomic analysis (Wang et al., 2020). Recently, glycan array assays have emerged as an excellent tool for high-throughput screening of the interaction between proteins and glycans (Malik et al., 2014; Nikiforova et al., 2022). By using of functionalized sugar nucleotide donors on glycan microarrays, a few plant glycosyltransferases have been characterized (Ruprecht et al., 2020). Moreover, nuclear magnetic resonance followed by computational modeling to study the 3D structure of LecRKs and glycans is another ascendant method to mine novel ligands. The newly developed RLP/KSeq technology is assisting us in rapidly identifying the novel immune receptors and helps to genetically map the genes responsible for ligand- triggered immune responses in plants (Lin et al., 2020).

### Signal transduction

As a kind of RLK protein kinase, autophosphorylation and transphosphorylation play important roles in LecRKs. At present, some LecRKs have been proven to function in autophosphorylation and some substrates have been identified. However, compared to the large number of LecRKs, these results only show the tip of the iceberg. Future researches should continue to focus on the identification of downstream substrates of LecRKs. Yeast two-hybrid and co-immunoprecipitation combined with MS assays are frequently-used means to identify the protein-protein interactions (PPIs). In addition, the recently developed proximity labeling (PL) approach has been used to screen PPIs. PL can detect weak, transient, hydrophobic, low-abundance and membrane-localized PPIs by exploring an unprecedented spatial and temporal protein interaction network in their native state (Yang et al., 2021). The application of PL methods may help to identify more interacting proteins of LecRKs in the future.

### Applications in crop improvement

LysM-RLKs control different stages of symbioses with beneficial mycorrhizal fungi and nitrogen-fixing bacteria (Chiu and Paszkowski, 2020). In addition to LysM-RLKs, a recent report has shown that G-type LecRK mediates the symbiotic interaction between *Populus* and the ectomycorrhizal fungus *Laccaria bicolor.* Moreover, genetic transformation of *PtLecRLK1* in *Arabidopsis* (Labbé et al., 2019) and switchgrass (Qiao et al., 2021; Shrestha et al., 2023) facilitated the colonization of *L. bicolor* with non-host plants. This work implied that LecRKs can be used to promote plant-microbe symbioses in crops. In addition to symbioses, LecRKs are also widely involved in resistance to pathogens and insects. Different LecRKs of different species might sense different ligands from multitudinous pathogens. Thus, transferring AtLORE to crops, such as rice, might enable the engineering of resistance traits to bacteria, such as *Xanthomonas* species, or constructing transgenic plants with chimeric LecRKs will achieve a potentially important step toward generating pathogen resistant crops (He et al., 2019). Meanwhile, LjLecRK of *Lotus japonicas*, plays important roles in freezing tolerance (Mustamin et al., 2023). SIT1 mediates ethylene production and salt-induced ethylene signaling (Li et al., 2014). These LecRKs can be used for engineering cold and salt-tolerant crops in the future.

## Author contributions

LL and NX drafted the manuscript. JL revised the manuscript. All authors contributed to the article and approved the submitted version.
