# Supplementary material for: Ligand recognition and signal transduction by lectin receptor-like kinases in plant immunity
Source: Front Plant Sci. 2023 Jun 15;14:1201805. doi: 10.3389/fpls.2023.1201805 (PMC10311507; doi:10.3389/fpls.2023.1201805)
Supplement: Supplementary file 1 [file DataSheet_1.pdf]

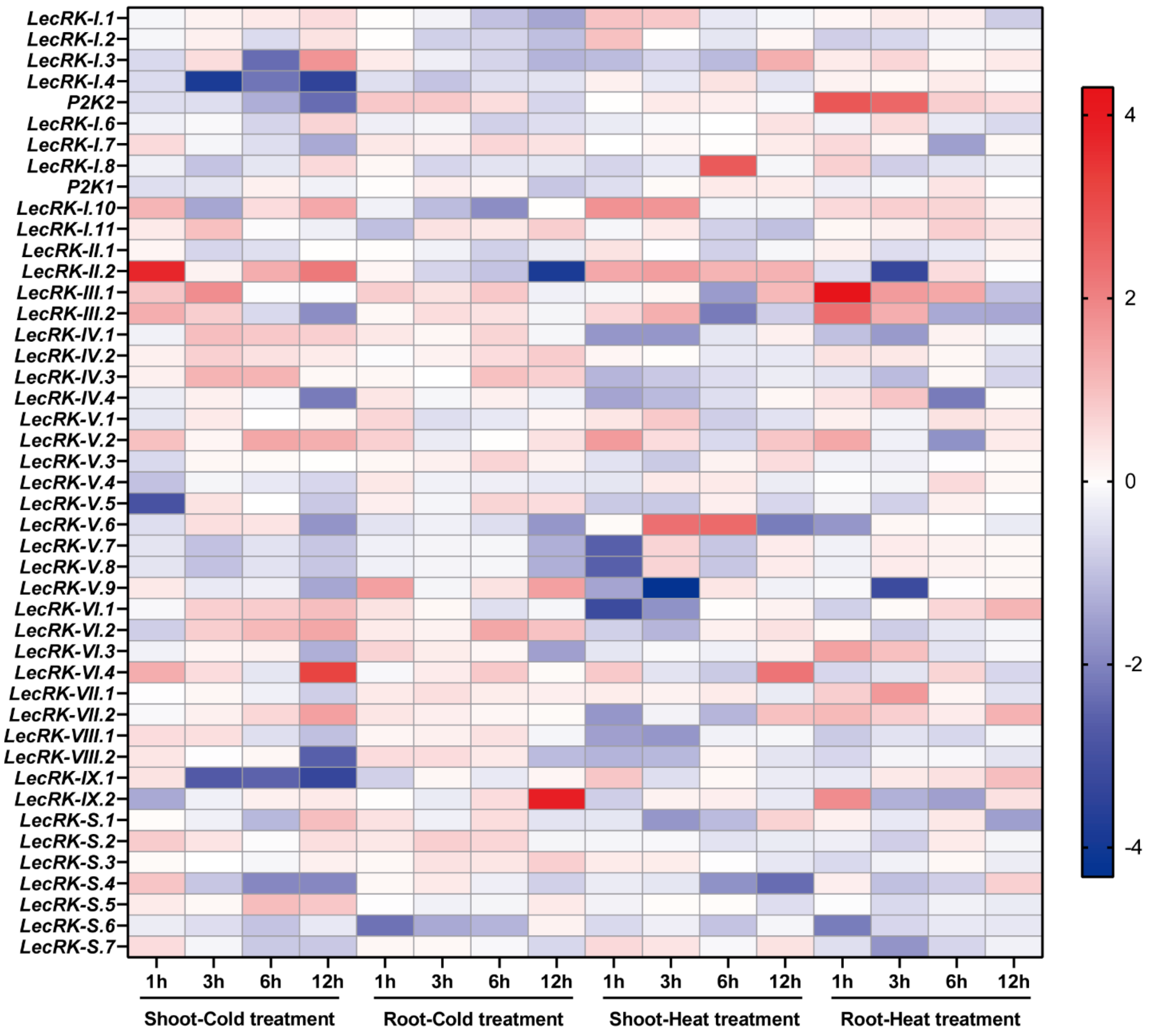

Figure S1. Expression profiles of *Arabidopsis thaliana* L-type *LecRKs* in response to cold (4℃) and heat (38℃) stresses. The expression data was obtained from Plant eFP database (http://bar.utoronto.ca/efp_arabidopsis/cgi-bin/efpWeb.cgi). The heatmap was plotted by GraphPad Prism 9. The color scale represent the relative expression values (Log_2Fold Change) of *LecRKs*.
